# The underlying mechanism behind the different outcomes of COVID-19 in children and adults

**DOI:** 10.3389/fimmu.2025.1440169

**Published:** 2025-04-30

**Authors:** Zifang Shang, Ling Huang, Shijie Qin

**Affiliations:** ^1^ Research Experiment Center, Meizhou People’s Hospital, Meizhou Academy of Medical Sciences, Meizhou, Guangdong, China; ^2^ Guangdong Engineering Technological Research Center of Clinical Molecular Diagnosis and Antibody Drugs, Meizhou People's Hospital, Meizhou, Guangdong, China; ^3^ Department of Critical Medicine, Shenzhen Clinical Research Centre for Geriatrics, Shenzhen People’s Hospital, The First Affiliated Hospital, Southern University of Science and Technology, Shenzhen, Guangdong, China; ^4^ Innovative Vaccine and Immunotherapy Research Center, The Second Affiliated Hospital Zhejiang University School of Medicine, Hangzhou, China; ^5^ Paediatric Research Institute, Shenzhen Children’s Hospital, Shenzhen, China

**Keywords:** COVID-19, children, adult, prognosis, immune response

## Abstract

Coronavirus disease 2019 (COVID-19), caused by SARS-CoV-2, has affected hundreds of millions of people globally, resulting in millions of deaths. During this pandemic, children have demonstrated greater resistance than adults, exhibiting lower infection rates, reduced mortality, and milder symptoms. Summarizing the differences in resistance between children and adults during COVID-19 can provide insights into protective mechanisms and potential implications for future treatments. In this review, we focused on summarizing and discussing the mechanisms for better protection of children in COVID-19. These protective mechanisms encompass several factors: the baseline expression of cell surface receptor ACE2 and hydrolase TMPRSS2, the impact of complications on COVID-19, and age-related cytokine profiles. Additionally, differences in local and systemic immune responses between children and adults also contribute significantly, particularly interferon responses, heterologous protection from non-COVID-19 vaccinations, and immune status variations influenced by micronutrient levels. The advantageous protection mechanisms of these children may provide insights into the prevention and treatment of COVID-19. Importantly, while age-related metabolic profiles and differential COVID-19 vaccine responses may contribute to protection in children, current comparative research remains limited and requires further investigation.

## 1 Introduction

The COVID-19 pandemic, caused by SARS-CoV-2, has had a profound global impact since its emergence. By 2025, confirmed cases worldwide have exceeded 700 million, resulting in over 6.5 million deaths (1). Studies have demonstrated that SARS-CoV-2 infection presents complex clinical manifestations, with marked differences across age groups (2, 3). A striking characteristic of COVID-19 is the marked disparity in disease presentation and severity between children and adults. While adults, especially the elderly, experience higher rates of severe illness and mortality, children generally demonstrate greater resistance to SARS-CoV-2, manifesting lower infection rates and milder symptoms (4–6). This age-related variation in disease outcomes represents a critical research focus. Elucidating the mechanisms behind children’s reduced susceptibility to severe SARS-CoV-2 infection could provide valuable insights into disease pathogenesis and guide therapeutic strategies. However, the underlying mechanisms for these age-dependent differences remain incompletely understood.

This review synthesizes the biological and immunological factors underlying the differential COVID-19 outcomes between children and adults. We examine seven key areas (1): clinical manifestations of COVID-19 in children versus adults; (2) age-dependent expression patterns of viral entry receptors and enzymes; (3) impact of comorbidities on disease severity; (4) age-related variations in immune responses, particularly cytokines and interferons; (5) Effects of micronutrients on disease outcomes; (6) contribution of heterologous immunity from childhood vaccinations; (7) age-specific differences in COVID-19 vaccine responses.

By systematically reviewing the above factors, we provide comprehensive insights into the mechanisms underlying milder COVID-19 manifestations in children compared to adults. These understandings are essential for optimizing age-specific COVID-19 management strategies and informing future epidemic preparedness and vaccine development. We also explore emerging research areas requiring further investigation, including age-related metabolic profiles (such as 1,5-Anhydro-D-glucitol), single-cell-scale molecular or immune repertoire, and trained innate immunity. Altogether, this review aims to advance our understanding of COVID-19 pathogenesis and identify novel therapeutic and preventive approaches.

## 2 Lower COVID-19 incidence and milder symptoms in children

Children (<18 years) present with diverse clinical manifestations when infected with SARS-CoV-2 (7–9). A European multi-center study of 582 children with COVID-19 (aged 0.5–12 years) reported fever in 65% of cases at presentation, with upper and lower respiratory tract symptoms occurring in 54% and 25% of cases, respectively (10). Additionally, gastrointestinal symptoms and headaches were reported in 22% and 28% of cases, respectively (10). A Chinese study of 171 SARS-CoV-2-positive children (0–15 years) documented cough (48.5%), sore throat (46.2%), and fever (41.5%) as predominant symptoms (11). Upon admission, 28.7% presented with tachypnea and 42.1% with tachycardia. Less common symptoms (< 10% of cases) included diarrhea, fatigue, rhinorrhea, vomiting, and nasal congestion (11). Generally, fever is the most common symptom in children with COVID-19, followed by cough, runny nose and sore throat, and some children also experience headache, diarrhea, vomiting, fatigue, myalgia, shortness of breath, tachycardia and rash (12). In a study of 149,082 COVID-19 patients with age information from the United States, symptoms in 2,572 (1.7%) children (<18 years old) were recorded. Among them, about 73% of infected children have fever, cough or shortness of breath, and 24% have sore throat, 28% have headache, 11% have nausea/vomiting and 13% have diarrhea (12). Notably, the proportion of these symptoms is significantly lower than that of the remaining adult patients aged 18-64 (12). Besides, as a strong predictor of positive SARS-CoV-2, loss of smell/hearing impairment is uncommon in children with COVID-19 (13, 14).

**Figure 1:**
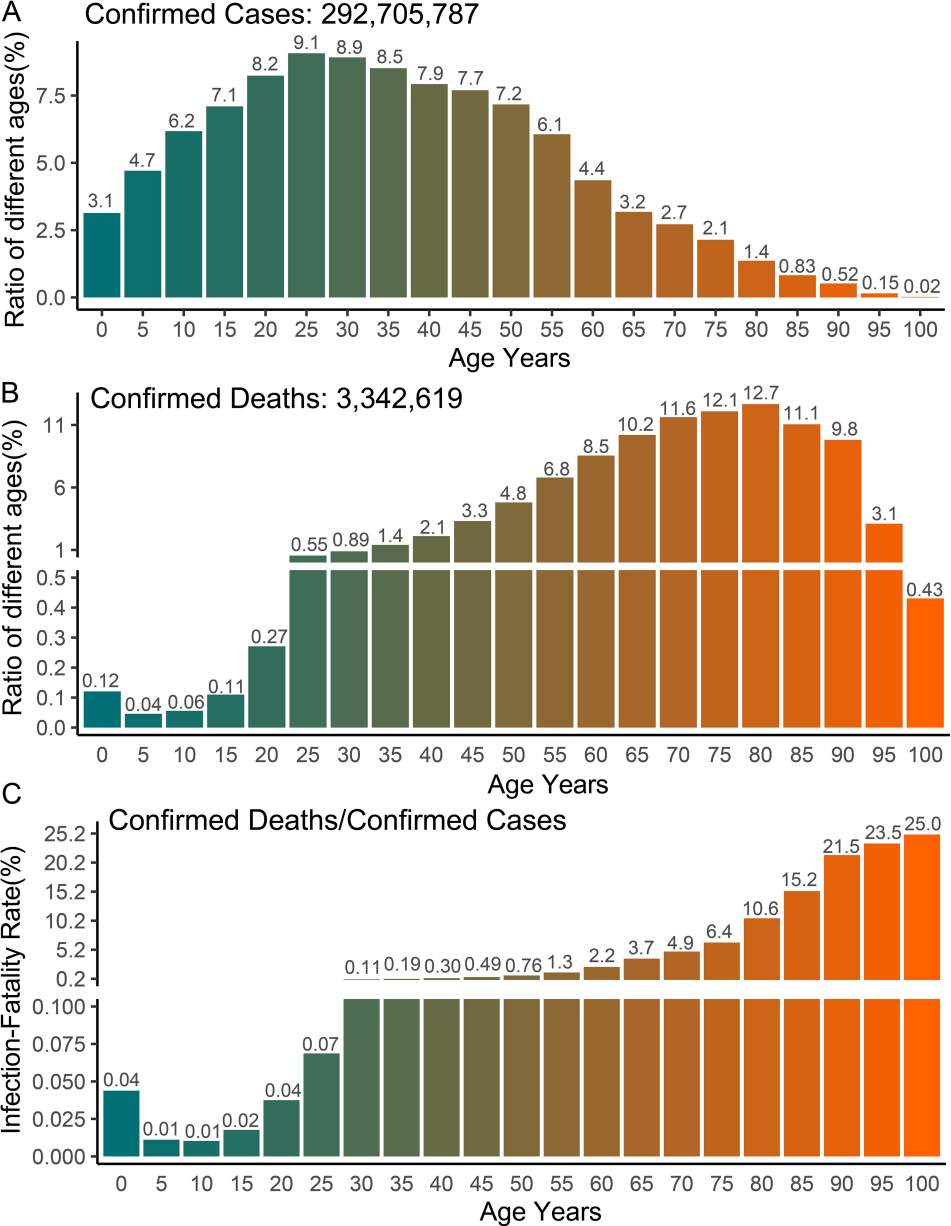
Global COVID-19 cases and deaths with age statistics. **(A–C)** The number of confirmed cases, deaths and infection fatality rate of COVID-19 in different age groups. The data shown in the graph includes the number of confirmed cases and deaths of COVID-19 with age records available in 77 countries. The original data comes from the COVerAGE-DB database (https://www.coverage-db.org/), which is an open-access database including cumulative counts of confirmed COVID-19 cases, deaths, tests, and vaccines by age and sex. In order to calculate infection fatality rate, only confirmed cases and deaths with age information were extracted for analysis.

The COVerAGE-DB database has documented hundreds of millions of confirmed cases and deaths of COVID-19 by age and gender, making it feasible to compare the prognosis between adults and children. Our analysis of age-stratified data from over 290 million confirmed cases and 3 million deaths reveals that children (<15 years) represent approximately 22% of confirmed COVID-19 cases (**Figure 1A**) but only 0.33% of COVID-19-related deaths (**Figure 1B**). Beyond population-adjusted analyses, the infection fatality rate in children (<15 years) demonstrated striking age-dependent differences: 8-fold lower than in adults aged 20–40 years, 81-fold lower than those aged 40–60 years, and 320-fold lower than individuals aged 65–80 years (**Figure 1C**). These data demonstrate children’s enhanced protection against severe COVID-19 outcomes relative to adults. Previous studies have reported that children (<18 years) represent less than 2% of global cases, typically experiencing asymptomatic or mild infections with rapid recovery without intervention (7–9). A Chinese cohort study of 70,117 COVID-19 cases (including 1,023 deaths) revealed markedly lower adjusted mortality rates in children compared to adults, with 0.0026% (0–9 years), 0.0148% (9–19 years), 0.060% (20–29 years), 0.1460% (30–39 years), 0.2950% (40–49 years), 1.25% (50–59 years), and 3.99% (60–69 years) (15).

Despite generally milder COVID-19 manifestations in children, significant age-dependent variations exist within pediatric populations. Our analysis reveals higher death rates and infection mortality in children under 5 years compared to those aged 5–15 years, suggesting poorer prognosis in younger children infected with SARS-CoV-2 (**Figures 1B, C**). Previous studies have also found that infants under 1 year of age have a higher risk of developing severe disease compared to older children (5, 6). Notable exceptions to mild pediatric cases include severe presentations and fatalities, primarily attributed to toxic shock-like syndrome or Multisystem Inflammatory Syndrome (MIS) (16–18). Children with MIS experience excessive pro-inflammatory responses or cytokine storms similar to adults, potentially progressing to acute respiratory distress syndrome (ARDS) (14, 16, 17). Specifically, the clinical presentation of MIS varies significantly among children of different age groups. Particularly in young children, MIS presents with more severe symptoms and a higher mortality rate. This condition is particularly pronounced in neonates, which is termed Multisystem Inflammatory Syndrome in Neonates (MIS-N) (16–18). MIS typically manifests 4 to 6 weeks after SARS-CoV-2 infection, characterized by persistent fever, mucocutaneous inflammation, lymphopenia, and elevated circulating acute phase reactants (C-reactive protein, IL-6, ferritin, and procoagulant factors) (14, 16, 17). Children with severe MIS may have hypotension, shock, myocarditis, myocardial dysfunction or/and coronary artery changes, which are the main culprits of severe symptoms and even death (14, 16, 17). The exact etiology of severe MIS remains unclear. There is a notable clinical overlap between MIS and Kawasaki disease, suggesting that SARS-CoV-2 infection may trigger Kawasaki-like inflammatory responses in susceptible children (16, 17). These age-dependent variations in disease severity may reflect developmental differences in immune system maturation, particularly in innate immune responses and cytokine regulation, which undergo significant changes during early childhood.

## 3 Children express lower ACE2 receptors and hydrolases than adults

**Figure 2:**
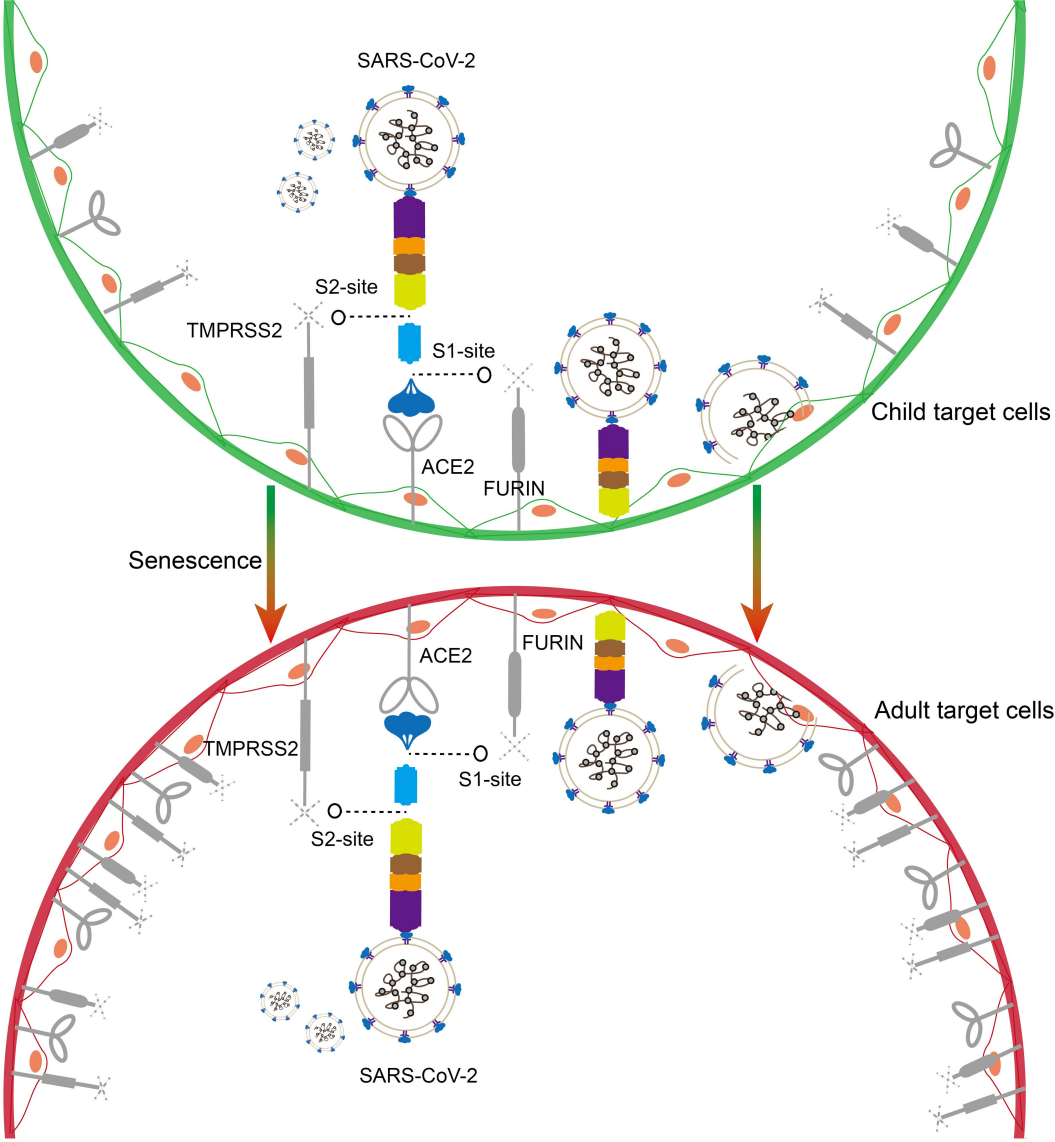
Schematic representation of SARS-CoV-2 entry into target cells. The SARS-CoV-2 spike protein binds to the ACE2 receptor and enters target cells with the assistance of the hydrolases TMPRSS2 and FURIN (children on the top, adults on the bottom). Higher baseline expression of ACE2 and TMPRSS2 and FURIN in the adult population may favor SARS-CoV-2 entry.

SARS-CoV-2 enters cells through endocytosis, a process initiated by fusion with the cell membrane (**Figure 2**) (19, 20). The SARS-CoV-2 spike (S) protein, which facilitates viral entry into cells, consists of two subunits, S1 and S2. Following binding to the ACE2 receptor, the S protein undergoes a conformational change required for activation and fusion (19). The conformational change of the S protein requires cleavage at two sites, S1 and S2, by the enzymes FURIN and TMPRSS2 (19). Consequently, baseline expression levels of ACE2, TMPRSS2, and FURIN influence SARS-CoV-2 cellular entry efficiency. Age-dependent differences in their expression may contribute to variations in infection susceptibility and viral load between children and adults (**Figure 2**).

Age-dependent expression of ACE2 has been reported in nasal epithelial cells at the primary site of infection, with the lowest expression in children under 10 years of age, and ACE2 expression increased with age in the 10-17, 18-24, and older age groups (21). Using single-cell sequencing and *in situ* hybridization techniques, Bryce et al. demonstrated lower TMPRSS2 expression in the lungs and respiratory epithelial cells of infants (2 years) and children (3–17 years) compared to adults (54–69 years). Their findings highlight TMPRSS2 as a potential therapeutic target for SARS-CoV-2 inhibition (22). Furthermore, research has shown that expression levels of ACE2 and TMPRSS2 are lower in full-term newborns compared to adults (23). However, some inconsistencies have been observed in studies examining age-related variations in TMPRSS2 and FURIN expression levels (24–26). While current evidence suggests lower baseline expression of ACE2, TMPRSS2, and FURIN in children compared to adults, more detailed investigations are needed to elucidate expression patterns across specific pediatric age groups.

The elevated expression of ACE2, TMPRSS2, and FURIN in adults can be partially attributed to factors such as smoking and chronic obstructive pulmonary disease (COPD) (27, 28). The absence of smoking history and healthier respiratory function in children likely contributes to their enhanced resistance to SARS-CoV-2 infection (27–29). Notably, two other coronaviruses, SARS-CoV and HCoV-NL63, also use ACE2 as a cellular receptor (30). The higher prevalence of HCoV-NL63 infections in adults compared to children suggests a similar age-dependent pattern of susceptibility across ACE2-utilizing coronaviruses (31). Nonetheless, these reported differences in SARS-CoV-2-related receptors or hydrolases may stem from differences in research methods, sample sizes, or the population being studied. When discussing the age difference of ACE2, TMPRSS2 and FURIN, it is crucial to consider the heterogeneity of subjects and the appropriate threshold range. Future studies need to divide the age groups more carefully to more strongly demonstrate the baseline differences of these key genes and their effects on the susceptibility to SARS-CoV-2.

## 4 Lower prevalence of comorbidities in children with COVID-19

A key clinical factor distinguishing pediatric from adult COVID-19 patients is the prevalence of comorbidities. Compared to children, many adult COVID-19 patients with severe outcomes or fatalities, have multiple comorbidities (32–34). Studies have demonstrated increased risk of severe disease and mortality in COVID-19 patients with underlying conditions, including hypertension, diabetes, coronary atherosclerosis, malignancies, obesity, COPD, and hepatic or renal dysfunction (35, 36). While adult COVID-19 patients are more likely to develop severe illness due to underlying diseases, children with chronic diseases also deserve special attention. For example, children with diabetes, obesity, or congenital heart disease may face a higher risk of severe disease (36–38). However, compared to adult patients, these pediatric patients still seem to have some protective advantages when facing COVID-19. This may be related to children’s overall stronger immune response capability and other age-related protective factors. Nevertheless, the mechanisms by which comorbidities promote susceptibility to COVID-19 and synergistically exacerbate the disease are still under investigation.

Comorbidities may enhance SARS-CoV-2 infection and replication through multiple mechanisms. For instance, patients with diabetes and certain cancers (particularly colon and lung) express higher levels of ACE2, which facilitates viral entry (37, 38). The pathological changes associated with lung cancer or COPD create additional vulnerabilities: tissue fibrosis, compromised mucosal barrier function, and a chronic inflammatory microenvironment collectively impair viral clearance in these patients (39, 40). Tong et al. identified 1,5-Anhydro-D-glucitol deficiency as a potential mechanism for increased SARS-CoV-2 susceptibility in diabetic patients (41). This small molecule metabolite interacts with specific sites (V952 and N955) on the S2 subunit of spike protein, modulating virus-cell membrane fusion (41). Notably, 1,5-Anhydro-D-glucitol levels decline with age, potentially contributing to age-dependent disease susceptibility. Hyperglycemia can compromise host defense mechanisms by altering multiple physiological pathways, which leads to increased plasma osmotic pressure, while simultaneously impairing leukocyte chemotaxis, reducing phagocytic activity, and diminishing intracellular pathogen killing capacity. These comprehensive alterations collectively weaken the host immune response to viral invasion (42). The widespread use of ACE inhibitors and ACE receptor blockers in adult hypertensive patients, but rarely in children, represents another age-specific factor. These medications typically increase ACE2 expression, potentially affecting viral entry (43, 44). Moreover, patients with comorbidities generally exhibit multiple pathophysiological alterations that may worsen COVID-19 outcomes, including compromised immunity, chronic inflammation, hypercoagulability, and impaired respiratory function (35, 45, 46).

## 5 Age-dependent differences in cytokine storm development

**Figure 3:**
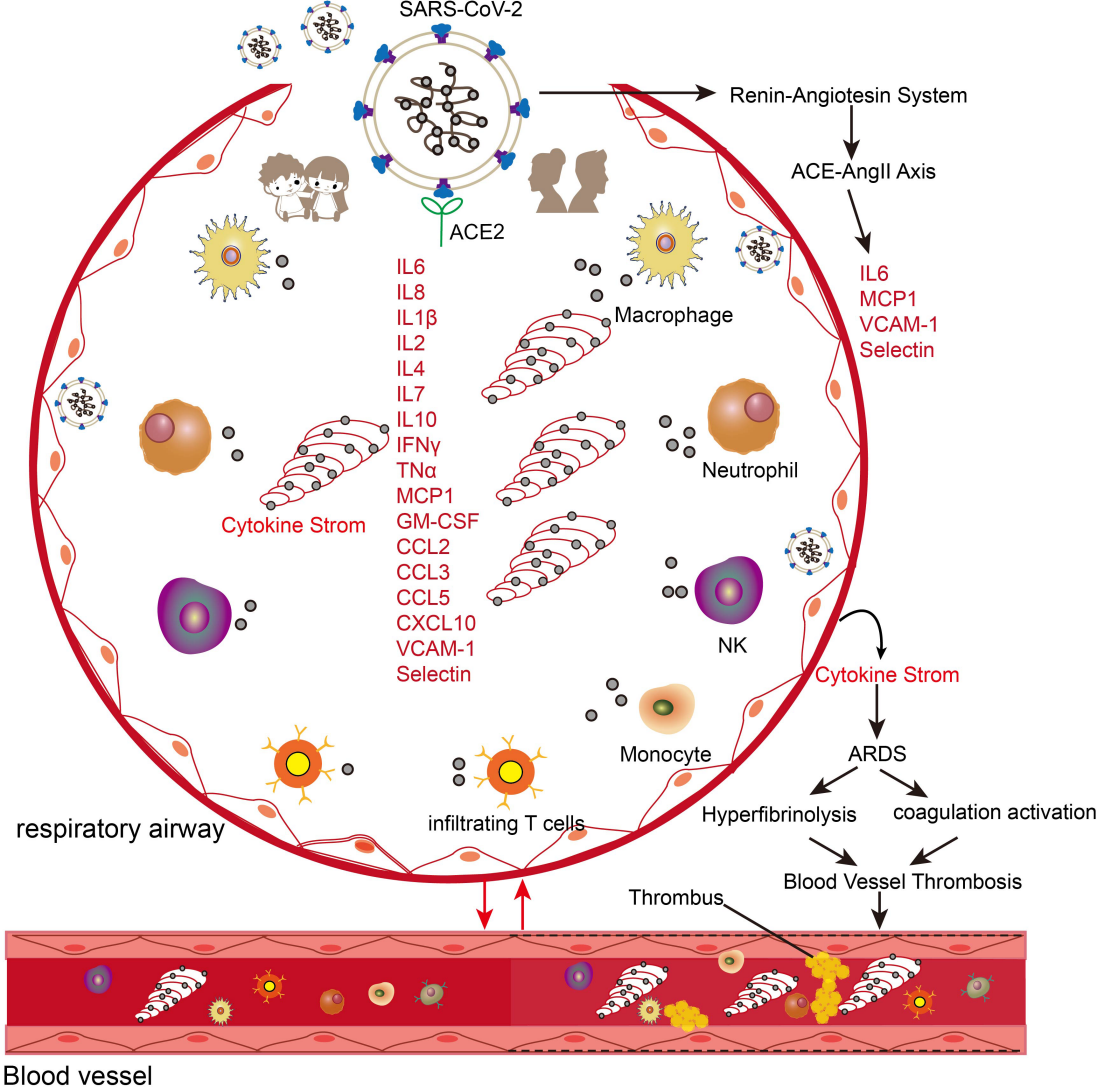
Schematic illustration of the effects of cytokine storm in COVID-19 patients. The entry of SARS-CoV-2 into the respiratory system triggers the migration and enrichment of a large number of innate and adaptive immune cells and triggers the activation of the ACE-AngII-Axis axis. The higher expression of cytokines and inflammatory genes in the adult population leads to crosstalk with the vascular and coagulation systems to trigger a series of acute respiratory syndromes (ARDS), coagulation systems, and hyperfibrinolysis.

Dysregulated cytokine responses, commonly referred to as cytokine storms, represent a major cause of mortality in severe COVID-19 cases (47, 48). While cytokines are essential mediators of immune responses to infections, their excessive production can trigger severe tissue damage and organ failure. Cytokine storms can increase vascular permeability, facilitating inflammatory mediator infiltration into tissues and amplifying the inflammatory response (**Figure 3**) (47). Additionally, these responses trigger substantial nitric oxide (NO) release, leading to vascular damage, endothelial dysfunction, coagulation abnormalities, and potentially septic shock (49). SARS-CoV-2 infection induces the production of multiple cytokines and chemokines, including pro-inflammatory mediators (IL-6, IL-1β, TNF-α), interferons (IFN-γ), interleukins (IL-2, IL-4, IL-7, IL-8, IL-10), and chemokines (MCP-1, GM-CSF, CCL2, CCL3, CCL5, CXCL10) (50) (**Figure 3**). While cytokine elevation also occurs in mild COVID-19 cases, the magnitude is substantially lower than in severe cases (51).

Age-dependent variations in cytokine profiles have been observed: children with COVID-19 exhibit early elevation of specific cytokines (CXCL10, GM-CSF, IL-17A, and IFN-γ) but maintain lower levels of TNF and IL-6 compared to adults (**Figure 3**) (52, 53). Children demonstrate more rapid interferon-related gene responses in both blood and nasal epithelial cells (54). However, pediatric cases progressing to severe COVID-19 show distinct immunological patterns: decreased T cell and natural killer cell populations, impaired interferon responses, and elevated IL-6 and IL-10 levels compared to mild cases (25, 55).

Aging is also associated with elevated baseline inflammatory cytokine levels in both healthy and diseased individuals, which may contribute to hypercoagulability and fibrinolytic dysfunction in elderly patients with COVID-19 (56). For example, interleukin-7 (IL-7), an age-associated cytokine, shows elevated expression in cardiac, pulmonary, and vascular tissues of elderly individuals and upregulates ACE2 expression in vascular endothelial cells through NF-κB-dependent mechanisms (57). The interaction between SARS-CoV-2 and ACE2 initiates a cascade of pathophysiological events (**Figure 3**). Viral binding activates the renin-angiotensin system, leading to increased angiotensin II (AngII) levels (58, 59). Elevated AngII correlates strongly with viral load and severity of lung injury in COVID-19 patients (58). The activated ACE2-AngII axis triggers multiple pathological processes, including enhanced cytokine production, macrophage infiltration, endothelial dysfunction, and coagulation abnormalities characterized by increased thrombin formation and impaired fibrinolysis (58, 60). SARS-CoV-2 infection not only triggers enhanced cytokine responses in aged individuals but also accelerates cellular aging processes in lung tissue (**Figure 3**) (61). This acceleration is evidenced by multiple molecular changes in COVID-19 patients compared to age-matched controls. The changes include upregulation of aging markers (p16, p21, p53) and aging-associated secretory factors such as IL-6, alongside enhanced DNA oxidative damage as indicated by elevated 8-OHdG levels. Additionally, these patients exhibit rapid depletion of nuclear architecture proteins, specifically lamin LAP2 and heterochromatin protein HP1g (61). These findings indicate a bidirectional promotion relationship between aging and SARS-CoV-2 infection, and age-related cytokines can serve as mediators in exacerbating the disease.

## 6 Immune superiority of children in response to SARS-CoV-2

**Figure 4:**
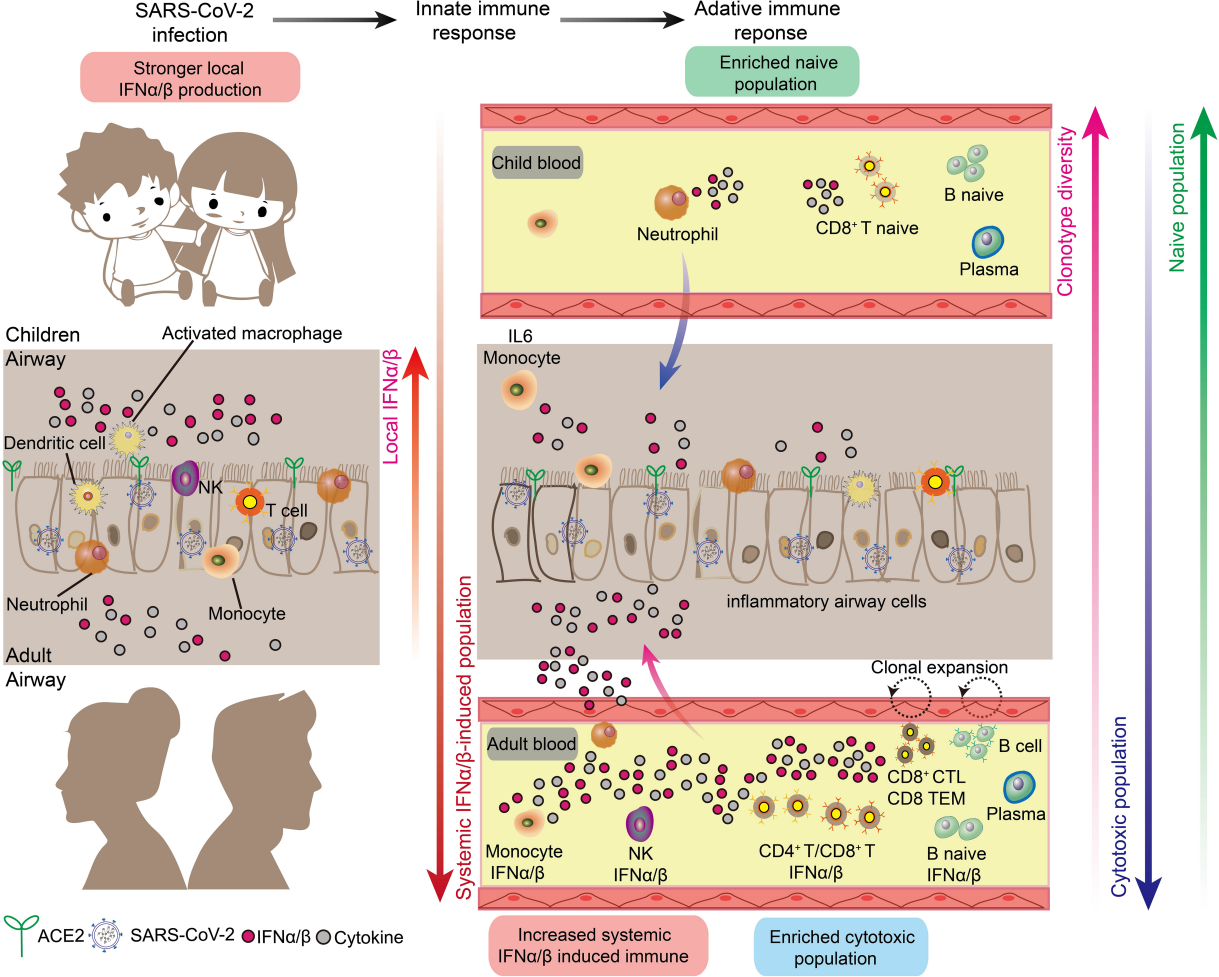
Respiratory and systemic responses to SARS-CoV-2 infection in children and adults. There are marked differences in the innate and adaptive immune responses in children and adults triggered by SARS-CoV-2 infection, as follows. 1. The local respiratory tract of children has a stronger interferon response and more comprehensive immune cells to suppress the downward spread of SARS-CoV-2. 2. Adults have a stronger systemic interferon response and systemic inflammatory storm than children. 3. Children have higher B-cell and T-cell clonal diversity than adults. 4. Children have higher naive immune cells than adults, and adults have higher induced cytotoxic immune cells.

Emerging evidence indicates that age-dependent variations in immune responses to SARS-CoV-2 contribute to more favorable outcomes in children (**Figure 4**). Paradoxically, children with COVID-19 generate lower levels of virus-specific antibodies, with approximately half the antibody titers observed in adults and reduced neutralizing activity (62, 63). Similarly, children exhibit diminished systemic T-cell responses compared to adults (64), despite experiencing milder disease. This apparent contradiction may be explained by children’s enhanced initial immune response, which provides rapid and effective pathogen clearance through robust innate immune mechanisms (65–67).

Children possess several distinct innate immune advantages in their response to viral infections (**Figure 4**). Most notably, children maintain higher baseline levels of immune cells critical for early antiviral responses in their nasal passages, contrasting with the relatively sparse immune cell populations found in healthy adults (24, 68). The enhanced presence of neutrophils in children’s nasal cavities facilitates rapid accumulation at infection sites and subsequent recruitment and activation of additional immune cell populations (24, 69). Environmental factors may further enhance children’s immunological advantage. Regular exposure to diverse pathogens in settings such as schools appears to strengthen nasal innate immunity. Recent research demonstrates that frequent encounters with viral and bacterial pathogens enhance children’s innate immune responses, potentially contributing to their protection against severe COVID-19 (70). This form of immune conditioning may partially explain children’s enhanced resistance to SARS-CoV-2 infection. Children exhibit enhanced baseline interferon responses in airway tissues and demonstrate more robust interferon induction by nasal immune cells following SARS-CoV-2 infection compared to adults (24, 67, 68). This heightened interferon response, coupled with pre-activated airway epithelial cells, enables rapid viral containment during early infection stages (71). Molecular analyses reveal that children’s enhanced innate immune responses correlate with elevated baseline expression of pattern recognition receptors, particularly MDA5 and RIG-I, in intranasal epithelial and immune cells. These receptors facilitate rapid recognition and binding of SARS-CoV-2 RNA (24). Additionally, children’s nasal tissues demonstrate an increased propensity to generate specialized T cell populations, including KLRC1 cytotoxic T cells and memory-phenotype CD8+ T cells, which contribute to both immediate viral clearance and long-term immunity (24). Collectively, these findings indicate that children’s immunological advantage against COVID-19 primarily stems from enhanced localized respiratory immune responses rather than systemic immunity.

The adaptive immune response to SARS-CoV-2 shows distinct age-dependent patterns, with children exhibiting reduced systemic inflammation compared to adults (**Figure 4**). Adult COVID-19 patients demonstrate more extensive systemic immune activation, characterized by increased numbers of circulating cytotoxic T lymphocytes, NK cells, and effector memory cells, alongside enhanced interferon responses, compared to infected children (64, 67, 68, 72). These findings indicate more widespread systemic immune and inflammatory responses in adult COVID-19 patients. Conversely, children’s circulation is characterized by a predominance of naive lymphocytes (68), correlating with reduced levels of circulating cytokines and inflammatory markers compared to adults (25, 55, 68). Furthermore, lymphopenia, a hallmark of severe COVID-19, occurs rarely in pediatric cases (73). The abundance of naive lymphocytes in children may provide dual protection: enhanced resistance to SARS-CoV-2-mediated cellular damage (including apoptosis, autophagy, thymic suppression, and bone marrow injury) (74, 75), and reduced inflammatory cell migration to the lower respiratory tract. In contrast, adults experience greater systemic inflammation, promoting migration of dendritic cells, monocytes, and lymphocytes from peripheral circulation to pulmonary tissues - a pattern consistent with radiological findings in severe cases (76, 77). Single-cell sequencing analyses have also revealed greater T-cell receptor (TCR) repertoire diversity in children with COVID-19, attributed to their higher proportion of naive immune cells. While adult COVID-19 patients demonstrate more restricted T cell clonal expansion (68). This pattern aligns with normal thymic developmental progression, wherein thymic function - essential for T cell development and TCR formation - gradually declines from fetal life through childhood to adulthood, resulting in reduced naive T cell production and consequently decreased TCR repertoire diversity (78, 79). Notably, the naive state of the immune system in children’s blood may provide a broader repertoire of immune receptors that can be used to detect various pathogens.

Obviously, the kinetics of immune cell responses significantly differ between children and adults, contributing to the disparity in COVID-19 outcomes. Children usually have a faster and stronger innate immune response to SARS-CoV-2 infection, characterized by rapid activation of neutrophils and natural killer (NK) cells, and quicker mobilization of interferon-producing cells, leading to earlier viral clearance (24, 67). This swift response often results in more effective early control of the virus. In contrast, adults tend to have a delayed but more prolonged innate immune response, potentially contributing to the extended inflammation observed in severe cases (24, 80). The adaptive immune response also shows age-related differences. Children exhibit a more diverse T cell receptor (TCR) repertoire, allowing for potentially broader recognition of viral antigens (68), while adults often display a more focused but potentially less flexible TCR repertoire (68). These differences in immune cell kinetics likely underpin the generally milder course of COVID-19 in children compared to adults.

Beyond the differences in T cell responses, recent investigations reveal distinct profiles in antibody functionality between children and adults with COVID-19. While children generate lower neutralizing antibody titers as previously noted (62, 63), they exhibit enhanced non-neutralizing antibody functions that may contribute to more effective viral clearance without excessive inflammation (62, 81). Bartsch et al. demonstrated that pediatric COVID-19 patients develop antibodies with superior Fc-mediated effector functions, including more potent antibody-dependent cellular cytotoxicity (ADCC) and complement activation, despite lower overall titers (81). This functional advantage in non-neutralizing antibody properties may partially explain children’s ability to resolve infection with milder symptoms.

Additionally, qualitative antibody differences appear significant, with children producing IgG with distinct glycosylation patterns that favor pathogen clearance while limiting inflammatory damage (62, 82). Pierce et al. found that pediatric patients maintain antibodies with broader epitope recognition beyond the spike receptor-binding domain, potentially offering more comprehensive immune coverage against viral variants (67). These findings suggest that children’s unique antibody profiles emphasize functional quality rather than quantity, potentially contributing to their reduced susceptibility to hyperinflammatory states observed in severe adult cases (62, 67). The mechanisms underlying these qualitative differences remain under investigation but may involve age-dependent variations in B cell development, selection, and maturation pathways (82).

Interestingly, although strong innate immunity provides children with better protection against SARS-CoV-2, it cannot explain their increased susceptibility to common respiratory syncytial virus and influenza virus (83, 84). This apparent contradiction may be elucidated through several interrelated factors. Primarily, while children generally possess a more active innate immune system, their adaptive immunity, which confers pathogen-specific protection, is still in the developmental stage, resulting in significantly weakened memory protection of T and B cells against common viruses compared to adults. This dichotomy may elucidate why they can effectively combat novel viruses like SARS-CoV-2, but remain susceptible to common respiratory pathogens. Secondarily, diverse viruses employ distinct mechanisms for infection and immune evasion. SARS-CoV-2 may be more vulnerable to innate immune responses, whereas RSV and influenza have evolved strategies to circumvent these initial defenses in children. Moreover, children’s frequent close interactions in school environments escalate their exposure to common respiratory viruses. Despite their robust innate responses, this increased exposure potentially overwhelms their developing immune systems. Concurrently, anatomical factors in children, such as smaller airways and incompletely developed respiratory structures, may contribute to their heightened susceptibility to respiratory infections, irrespective of immune response intensity. Finally, compared to common viruses, SARS-CoV-2 has been reported to exhibit significant immune sin against T and B cells, leading to a decrease in adaptive immune protection in adults, which can be more effectively compensated for by the innate immune system (85–87).

## 7 Age-dependent variations in micronutrient status and COVID-19 outcomes

Immune system dysfunction resulting from poor diet and nutritional deficiencies was a major risk factor for respiratory viral infections (88). Optimal nutritional status can provide the appropriate micronutrients to ensure the best bodily response for preventing infection (88). Research has shown that children generally have higher baseline levels of vitamin D and zinc compared to adults (89, 90). Moreover, vitamin D and zinc deficiency were often found to correlate with COVID-19 severity (89, 90). Here, we focus on summarizing the changes in vitamin D and zinc in COVID-19 and their potential mechanisms affecting the progression of COVID-19.

Geographic analyses examining relationships between soil mineral content and COVID-19 mortality revealed that regions with lower soil zinc concentrations demonstrated significantly higher COVID-19 case fatality rates in the United States (91). In addition, multiple studies have reported that low zinc levels are associated with advanced age, as well as higher ICU admission rates, severity rates and higher clinical complications in COVID-19 patients (92, 93). Zinc supplementation is believed to be helpful in COVID-19 treatment (94, 95). Some studies have indeed demonstrated that zinc or zinc compounds have nanomolar affinity with the main protease (Mpro) of SARS-CoV-2 and can stably form an inhibited Mpro-Zn^2+^ complex to inhibit virus replication and activity (96, 97). Zn affects multiple aspects of the immune system ranging from the skin’s barrier to lymphocytes. For example, Zn deficiency prevents T lymphocytes from growing and functioning, as well as disrupt macrophage killing, cytokine production and phagocytosis (98). Moreover, Zn is a component of many metalloenzymes, enzyme activator or immunoglobulin G (IgG) (98). Particularly, as the core component of plenty of zinc finger proteins, Zn can ensure that zinc finger antiviral proteins correctly recognize, bind, cleave or degrade RNA viruses (98, 99). Zn also has powerful antioxidant properties that fight free radicals and prevent diseases caused by oxidative stress (98). More importantly, Zn can inhibit the excessive immune response, because Zn deficiency can lead to excessive immune response and cytokine storm in the immune system (100).

Vitamin D represents one of the most extensively investigated micronutrients in COVID-19 pathogenesis. Studies have consistently demonstrated associations between vitamin D deficiency and increased disease severity, particularly regarding ICU rates among elderly patients over 70 years (101, 102). Retrospective analyses have revealed that children with COVID-19 exhibit lower vitamin D levels compared to healthy controls, with levels inversely correlating with fever severity (88). Furthermore, vitamin D status has emerged as a potential predictive biomarker for MIS in children (103). Strong correlations between vitamin D status and cytokine storm severity in COVID-19 patients suggest that vitamin D deficiency may contribute significantly to mortality risk (104, 105). Beyond its classical role in calcium-phosphorus metabolism and bone homeostasis, vitamin D demonstrates important immunomodulatory functions (105). Vitamin D can enhance innate cellular immunity by promoting antimicrobial peptide production and subsequent antiviral responses (105). It demonstrates dual immunomodulatory effects: suppressing proinflammatory cytokine production (IFNγ, IFNβ, TNFα, and IL6) while enhancing regulatory cytokine IL-10 expression, thereby mitigating cytokine storm risk (106). In respiratory syncytial virus-infected lung epithelial cells, vitamin D upregulates IKBα, an NF-κB inhibitor, resulting in reduced expression of inflammatory mediators such as IFN-β and CXCL10 (107). Clinical studies demonstrate that vitamin D supplementation significantly reduces COVID-19 mortality, with an observed 2.14-fold decrease in death rates (104, 108, 109). This protective effect appears to operate through multiple mechanisms, including modulation of INOS1, IL1β, IFNγ and ICAM1 expression (104, 108). Additionally, vitamin D maintains pulmonary homeostasis by inhibiting TGF-β-mediated fibrosis, protecting alveolar epithelial cells from apoptosis, and regulating the renin-angiotensin axis to preserve lung epithelial integrity (104).

The pleiotropic effects of vitamin D collectively promote lung epithelial homeostasis and facilitate ARDS resolution (110). The evidence demonstrates that higher baseline levels of vitamin D and zinc, typically observed in children compared to adults, contribute to improved COVID-19 outcomes through multiple mechanisms: enhanced immune function, reduced inflammatory responses, and potential direct antiviral effects. These micronutrient-associated advantages may partially explain the less severe disease course and better recovery patterns observed in pediatric populations.

## 8 Childhood vaccination-induced heterologous protection against COVID-19

Vaccines are fundamental to viral disease prevention, generating durable immunity through memory T and B cell induction. Vaccination coverage differs significantly between pediatric and adult populations. Most national immunization programs mandate childhood vaccines, including two doses of the Measles-Mumps-Rubella (MMR) vaccine before age 5 and Bacille Calmette-Guérin (BCG) vaccination (108). Previous research has demonstrated that tuberculosis vaccination confers protection against diverse viral pathogens, including both DNA and RNA viruses such as herpes and influenza viruses - a phenomenon known as heterologous immune protection (108). This protection mechanism involves immune responses to specific antigens triggered by prior exposure to unrelated antigens (111). Various childhood vaccines, including MMR, BCG, oral polio, diphtheria, pertussis, tetanus, and influenza vaccines, demonstrate immunological effects beyond their targeted pathogens (111).

This heterologous protection extends to COVID-19, with multiple studies documenting reduced infection rates and symptom severity among recipients of routine childhood vaccines, particularly the MMR and Tetanus-Diphtheria-Pertussis (Tdap) vaccines. Epidemiological and retrospective analyses of global data reveal a notable inflection point in COVID-19 severity around age 50 (111). This age-dependent pattern correlates with the historical implementation of mass measles-rubella vaccination programs approximately 50 years ago. Individuals over 50, who typically missed childhood MMR vaccination, demonstrate more severe COVID-19 outcomes compared to younger vaccinated cohorts. Moreover, a statistical method called overlap propensity score weighting was applied to more than 75,000 subjects and found that the hospitalization rate of patients previously vaccinated with MMR and Tdap decreased by 38% and 23%, and the ICU/death ratio decreased 32% and 20% (106). In addition, studies have reported that influenza vaccination is associated with a significant reduction in the risk of SARS-CoV-2 infection and the severity of COVID-19 (112). In particular, a study of more than 30,000 health workers in Qatar demonstrated that the influenza vaccine can not only reduce the risk of SARS-CoV-2 infection, but also significantly achieve 90% efficacy in severe COVID-19 protection [98]. An Italian study revealed that subjects vaccinated with both quadrivalent seasonal influenza vaccine and pneumococcal vaccine had a 58% higher neutralizing titer against SARS-CoV-2 than unvaccinated subjects, whereas it was 42% higher in subjects vaccinated with quadrivalent influenza vaccine alone (113).

The protective mechanisms of heterologous immunity may involve trained innate immunity or/and improved adaptive immunity of T/B cells (bystander activation and cross-reactivity) (113). Trained innate immunity hypothesizes that innate immune cells may be primed upon encountering exogenous or endogenous stimuli, leading to long-term metabolic and epigenetic reprogramming and to enhanced responses to a second challenge (113). Bystander activation refers to a heterologous response exerted by adjacent but unrelated T cells with different specificities. These allogeneic T cells may be activated by cytokines as a result of cellular activation during the classical response (113). In contrast, the cross-reactivity theory suggests that participating T cells may cross-react with antigens that exhibit some degree of amino acid similarity (113).

Specifically, the protective mechanism of influenza vaccination is relatively clear. Firstly, the seasonal influenza vaccination was found to induce trained immunity against SARS-CoV-2 (114). The authors observed increased cytokine production following stimulation of peripheral blood mononuclear cells with influenza vaiccine and BCG in a well-established model. Re-stimulation of these cells with inactivated SARS-CoV-2 induced higher IL-1RA (IL-1 receptor antagonist) and reduced proinflammatory IL-1β and IL-6 (114). Furthermore, Almazán et al. confirmed that influenza vaccine induced cross-reactive and cross-protective antibodies against SARS-CoV-2. They found that the small peptide NGVEGF/NGVKGF contained in the immunodominant region of neuraminidase of the H1N1 strain was also present in the most critical part of the S protein receptor binding domain (RBD) of SARS-CoV-2 (N481–F486) (115). The authors also identified 11 additional CD8^+^ cellular peptides that may cross-react with SARS-CoV-2 and influenza viruses, which protect approximately 40-71% of individuals from SARS-CoV-2 (115). In addition, Pallikkuth et al. partially demonstrated a bystander activation mechanism and found that the CD4^+^ response to H1N1 was strongly positively correlated with SARS-CoV-2-specific CD4^+^ cells. In their health care worker cohort, H1N1 antigen-specific CD4^+^ cells were present in SARS-CoV-2 positive (92%) and negative (76%) subjects, respectively (116).

Protection in COVID-19 by MMR or Tdap vaccines goes beyond induction of a trained innate immune. Studies have observed that some antigenic T-cell receptors that respond to SARS-CoV-2 proteins (Spike and Nucleocapsid) in patients with COVID-19 were the same as the antigenic receptors that respond to MMR and Tdap proteins (117). This suggests the existence of specific immunity that responded identically to SARS-CoV-2 antigens as well as to MMR and Tdap vaccine antigens. In addition, there are homologous sequences between the S protein of SARS-CoV-2 and the fusion protein F1 of measles virus and the envelope protein E1 of rubella, and they seem to have epitope properties (118), which suggests that receiving measles-rubella vaccine may also develop an immune response to SARS-CoV-2 infection. Gold et al. found that asymptomatic patients with COVID-19 had the highest IgG titers for mumps antibodies, whereas severe COVID-19 cases had very low IgG levels (119). Finally, the pre-existing T cells induced by common coronavirus (HCoV-HKU1, HCoV-OC43) can cross-recognize the internal proteins (nucleocapsid and ORF1) of SARS-CoV-2 (120, 121).

In summary, epidemiological observations of COVID-19 suggest that vaccine-induced heterologous immunity may be effective in preventing SARS-CoV-2 infection or at least severe disease. In the past decades of rapid development of vaccines, many countries have launched a large number of vaccination programs, especially for children. As a result, children have significantly more coverage of non-COVID-19 vaccines than adults, such as BCG, MMR and Tdap. It is reasonable to conclude that the heterologous immune protection against SARS-CoV-2 conferred by these widely administered childhood vaccines has become a significant focus of research interest.

Age-stratified analyses suggest differential associations between childhood vaccination status and COVID-19 outcomes. Epidemiology studies have examined the relationship between BCG vaccination policies and COVID-19 mortality rates across countries, with several showing lower COVID-19 case fatality rates in nations with universal BCG vaccination programs (122, 123). Miller et al. reported that countries without universal BCG policies (Italy, Netherlands, USA) had higher COVID-19 mortality compared to countries with long-standing universal BCG policies (124). However, these studies are complex and limited by potential confounding factors.

More rigorous investigations have attempted to control for confounding variables. A retrospective cohort study by Szigeti et al. analyzed over 6,000 healthcare workers and found that BCG vaccination was associated with a lower prevalence of COVID-19 diagnosis (125). Similarly, a matched case-control study by Sharma et al. suggested that MMR vaccination history correlated with reduced severity of COVID-19 symptoms, with the effect more pronounced in younger individuals who had more recent MMR administration (126). These findings align with previous studies on trained immunity, where Netea et al. demonstrated that BCG vaccination induces metabolic and epigenetic changes in innate immune cells, potentially enhancing responses to unrelated pathogens (113, 127).

The timing of vaccination appears potentially significant, with some evidence that vaccines administered during childhood may establish more durable heterologous protection. O’Neill and Netea proposed that early-life immune imprinting during critical developmental windows creates broader and more adaptable trained immunity responses (128). This concept is supported by experimental models showing that early-life BCG vaccination generates distinct epigenetic signatures compared to vaccination in adulthood (129).

These findings complement our understanding of children’s immunological advantages against COVID-19. The heterologous protection from routine childhood vaccinations may work synergistically with enhanced innate responses, favorable immune cell distributions, and reduced comorbidities to contribute to the reduced COVID-19 severity observed in pediatric populations (54, 122). This multi-layered protection underscores the importance of childhood immunization programs not only for targeted diseases but potentially for emerging pathogens as well.

## 9 Conclusions

Evidence consistently demonstrates higher SARS-CoV-2 infection rates and disease severity in adults, particularly the elderly, compared to children. The predominantly asymptomatic or mild presentation in pediatric populations offers unique insights into protective mechanisms against severe COVID-19. The reduced disease severity and infection rates in children could be attributed to several key mechanisms (**Table 1**). Primary among these is the enhanced innate immune response in the pediatric respiratory tract, characterized by abundant immune cells and rapid interferon responses. High expression of pattern recognition receptors (including MDA5 and RIG-I) in children’s nasal cavities facilitates rapid SARS-CoV-2 recognition and early immune activation, effectively limiting viral spread within the respiratory tract. A second critical factor is the relative absence of age-related comorbidities in children. Adults frequently present with conditions such as smoking-related lung damage, hypertension, lung cancer, and COPD, resulting in compromised respiratory function and endothelial-coagulation system dysfunction. These conditions are associated with airway cell dysregulation, leading to excessive inflammation and mucus production that exacerbates dyspnea and prolongs recovery in adult COVID-19 patients. Third, age-dependent metabolic factors play a significant role, exemplified by 1,5-Anhydro-D-glucitol, which demonstrates anti-SARS-CoV-2 activity and is notably depleted in elderly and diabetic patients. The potential contribution of other age-related metabolites warrants further investigation. Fourth, elderly individuals exhibit both immunosenescence and elevated baseline inflammatory states, characterized by higher cytokine levels, predisposing them to dysregulated immune responses and cytokine storms upon infection. Fifth, children typically maintain higher levels of essential micronutrients, particularly vitamin D and zinc, supporting optimal immune function and antiviral protein activity. This observation suggests potential therapeutic benefits of micronutrient supplementation in nutritionally deficient adult patients. Sixth, broader vaccination coverage in pediatric populations, including BCG, MMR, and influenza vaccines, may confer enhanced heterologous protection against SARS-CoV-2, an advantage often absent in adult populations. While age-dependent variations in ACE2, TMPRSS2, and FURIN expression may influence infection susceptibility and disease manifestation, current evidence remains inconclusive. Future investigations should establish appropriate expression thresholds and analyze well-defined population subgroups to better understand their contribution to COVID-19 severity.

**Table 1:** Mechanism summary of the better protection for children infected with SARS-CoV-2 than adults.

Difference factors	Adults	Children
Expression of ACE2, TMPRSS2, and FURIN	**↑**	**↓**
Respiratory system disorder caused by complications	**↑**	**↓**
Endothelial and Coagulation Disorders	**↑**	**↓**
Serum small molecule metabolite inhibiting spike protein	**↓** (1,5-Anhydro-D-glucitol)	**↑**
Baseline levels of senescence related cytokines	**↑**	**↓**
Respiratory immune cell abundance	**↓**	↑ (Innate immune cells)
Expression of pattern recognition receptors (MDA5 and RIG-I)	**↓**	**↑**
Response time and intensity of respiratory interferon	**↓**	**↑**
Systemic inflammation and interferon response	**↑**	**↓**
Distribution difference of immune repertoire	**↑** (Cytotoxic T cells and B cells)	**↑** (Naive T cells and B cells)
Distribution of micronutrients Vitamin D and Zinc	**↓**	**↑**
Heterologous immune protection of non-COVID-19 vaccine	**↓**	**↑** (BCG, MMR, Tdap, SIV)

The symbols ↑ and ↓ in represent increase and decrease, respectively.

Beyond these factors, age-dependent variations in COVID-19 vaccine responses may contribute to enhanced protection in children, contrasting with potentially suboptimal responses in older populations (121, 122). Given the widespread implementation of COVID-19 vaccination programs, understanding age-specific variations in vaccine responses becomes crucial for interpreting mortality and symptom patterns. The established pattern of enhanced vaccine responses in children, documented for various vaccines (54, 130, 131), may extend to COVID-19 vaccines. However, comparative data on age-specific COVID-19 vaccine responses remains limited. Future research priorities should focus on comprehensive analysis of age-dependent variations in antibody production and persistence, T cell response profiles, and long-term protective efficacy. These systematic investigations will be essential for informing and optimizing age-specific vaccination strategies.

Despite the generally milder COVID-19 manifestations in children, maintaining protective measures for pediatric populations remains essential. This necessity is underscored by the risk of severe complications, particularly MIS, in vulnerable groups such as infants and newborns. Furthermore, the continual emergence of new SARS-CoV-2 variants, combined with reduced protective measures, could lead to increased pediatric infection rates, straining healthcare resources and potentially increasing mortality. The concept of ‘immune debt’ requires careful consideration, as evidenced by recent surges in respiratory syncytial virus, influenza, and bacterial infections among children (132–135). While public health measures implemented during the pandemic effectively reduced SARS-CoV-2 transmission, the associated reduction in social interaction, physical activity, and routine vaccinations may have created unintended consequences, including compromised immune development, mental health challenges, and delayed disease detection. The accumulated impact of these factors over several years may manifest as significant health challenges if not properly addressed through comprehensive pediatric health policies. Public health strategies must balance protective measures against COVID-19 with interventions to prevent immune system development deficits, ensuring optimal long-term health outcomes for children.
